# Towards Clinical Application of Artificial Intelligence in Ultrasound Imaging

**DOI:** 10.3390/biomedicines9070720

**Published:** 2021-06-23

**Authors:** Masaaki Komatsu, Akira Sakai, Ai Dozen, Kanto Shozu, Suguru Yasutomi, Hidenori Machino, Ken Asada, Syuzo Kaneko, Ryuji Hamamoto

**Affiliations:** 1 Cancer Translational Research Team, RIKEN Center for Advanced Intelligence Project, 1-4-1 Nihonbashi, Chuo-ku, Tokyo 103-0027, Japan; hidenori.machino@riken.jp (H.M.); ken.asada@riken.jp (K.A.); sykaneko@ncc.go.jp (S.K.); 2 Division of Medical AI Research and Development, National Cancer Center Research Institute, 5-1-1 Tsukiji, Chuo-ku, Tokyo 104-0045, Japan; adozen@ncc.go.jp (A.D.); kshozu@ncc.go.jp (K.S.); 3 Artificial Intelligence Laboratory, Research Unit, Fujitsu Research, Fujitsu Ltd., 4-1-1 Kamikodanaka, Nakahara-ku, Kawasaki, Kanagawa 211-8588, Japan; akira.sakai@fujitsu.com (A.S.); yasutomi.suguru@fujitsu.com (S.Y.); 4 RIKEN AIP—Fujitsu Collaboration Center, RIKEN Center for Advanced Intelligence Project, 1-4-1 Nihonbashi, Chuo-ku, Tokyo 103-0027, Japan; 5 Biomedical Science and Engineering Track, Graduate School of Medical and Dental Sciences, Tokyo Medical and Dental University, 1-5-45 Yushima, Bunkyo-ku, Tokyo 113-8510, Japan

**Keywords:** ultrasound imaging, artificial intelligence, machine learning, deep learning, preprocessing, classification, detection, segmentation, explainability

## Abstract

Artificial intelligence (AI) is being increasingly adopted in medical research and applications. Medical AI devices have continuously been approved by the Food and Drug Administration in the United States and the responsible institutions of other countries. Ultrasound (US) imaging is commonly used in an extensive range of medical fields. However, AI-based US imaging analysis and its clinical implementation have not progressed steadily compared to other medical imaging modalities. The characteristic issues of US imaging owing to its manual operation and acoustic shadows cause difficulties in image quality control. In this review, we would like to introduce the global trends of medical AI research in US imaging from both clinical and basic perspectives. We also discuss US image preprocessing, ingenious algorithms that are suitable for US imaging analysis, AI explainability for obtaining informed consent, the approval process of medical AI devices, and future perspectives towards the clinical application of AI-based US diagnostic support technologies.

## 1. Introduction

Ultrasound (US) imaging is superior to other medical imaging modalities in terms of its convenience, non-invasiveness, and real-time properties. In contrast, computed tomography (CT) has a risk of radiation exposure, and magnetic resonance imaging (MRI) is non-invasive but costly and time-consuming. Therefore, US imaging is commonly used for screening as well as definitive diagnosis in numerous medical fields [1]. Current advances in image rendering technologies and the miniaturization of ultrasonic diagnostic equipment have led to its use in point-of-care testing in emergency medical care, palliative care, and home medical care [2]. It is worth considering the combination of US diagnostic capabilities and laboratory tests as the multi-biomarker strategy for prediction of clinical outcome [3]. However, US imaging exhibits characteristic issues relating to image quality control. In CT and MRI, image acquisition is performed automatically with a specific patient, a fixed measurement time, and consistent image settings. On the other hand, US imaging is acquired through manual sweep scanning; thus, its image quality is dependent on the skill levels of the examiners [4]. Furthermore, acoustic shadows owing to obstructions such as bones affect the image quality and diagnostic accuracy [5]. Certain US diagnostic support technologies are required to resolve these practical difficulties that arise in normalizing sweep scanning techniques and image quality.

In recent years, artificial intelligence (AI), which includes machine learning and deep learning, has been developing rapidly, and AI is increasingly being adopted in medical research and applications [6,7,8,9,10,11,12,13,14,15,16]. Deep learning is a leading subset of machine learning, which is defined by non-programmed learning from a large amount of data with convolutional neural networks (CNNs) [17]. Such state-of-the-art technologies offer the potential to achieve tasks more rapidly and accurately than humans in particular areas such as imaging and pattern recognition [18,19,20]. In particular, medical imaging analysis is compatible with AI, where classification, detection, and segmentation used as the fundamental tasks in AI-based imaging analyses [21,22,23]. Furthermore, many AI-powered medical devices have been approved by the Food and Drug Administration (FDA) in the United States [24,25].

The abovementioned clinical issues have affected and slowed the progress of medical AI research and development in US imaging compared to other modalities [26,27]. Table 1 shows the AI-powered medical devices for US imaging that have been approved by the FDA as of April 2021 (https://www.accessdata.fda.gov/scripts/cdrh/cfdocs/cfpmn/pmn.cfm, the access date was 10 May 2021) (Table 1). Deep learning requires the availability of sufficient datasets on both normal and abnormal subjects for different diseases in high-quality controls. It is necessary to assess the input data quality and to accumulate robust technologies, including effective data structuring and algorithm development, to facilitate the clinical implementation of AI devices. Another concern is the AI black box problem, whereby the decision-making process of the manner in which complicated synaptic weighting is performed in the hidden layers of CNNs is unclear [28]. Examiners need to understand and explain the rationale for diagnosis to patients objectively for obtaining informed consent in constructing valid AI-based US diagnostic technologies in clinical practice.

This review introduces the current efforts and trends of medical AI research in US imaging. Moreover, future perspectives are discussed to establish the clinical applications of AI for US diagnostic support.

## 2. US Image Preprocessing

US imaging typically exhibits low spatial resolution and numerous artifacts owing to ultrasonic diffraction. These characteristics affect not only the US examination and diagnosis but also AI-based image processing and recognition. Therefore, several methods have been proposed for US image preprocessing which eliminates noises that are obstacles to accurate feature extraction before US image processing. In this session, we present two representative methods: US image quality improvement and acoustic shadow detection.

Firstly, various techniques have been developed for US image quality improvement at the time of image data acquisition by reducing speckle, clutter, and other artifacts [29]. Real-time spatial compound imaging using ultrasonic beam steering of a transducer array to acquire several multiangle scans of an object has been presented [30]. Furthermore, harmonic imaging using endogenously generated low frequency to reduce the attenuation and improve the image contrast was proposed [31]. Several methods for US image enhancement using traditional image processing have been reported [32]. Despeckling is the representative research subject on filtering or removing punctate artifacts in US imaging [33]. In this method, the cause of the image quality degradation is eliminated during the US image generation phase or the noise characteristics are modeled along with the US image generation process following close examination. Current approaches for US image quality improvement using machine learning or deep learning include methods for improving the despeckling performance [34,35], and enhancing the overall image quality [36]. Such data-driven methods offer the significant advantage that it is not necessary to create a model for each domain. However, substantial training data with targeted high quality are required to improve the US image quality, and because the preparation of such a dataset is generally difficult, critical issues arise in clinical application.

**Figure 1:**
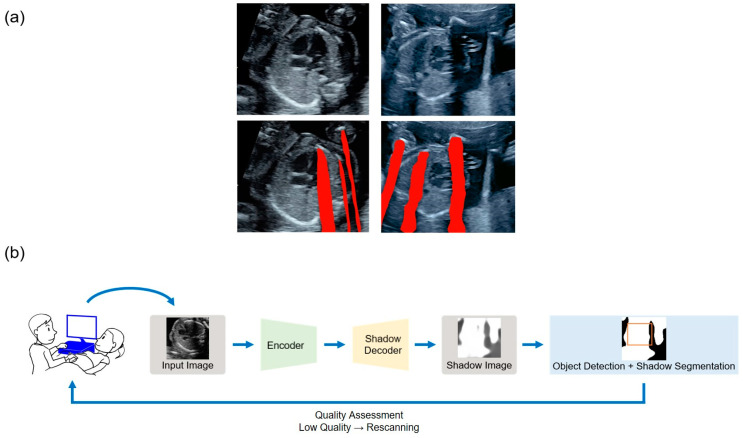
Acoustic shadow detection: (**a**) The red areas represent the segmented acoustic shadows using the semi-supervised approach [43]. (**b**) As a candidate for clinical application, examiners can evaluate whether the current acquired US imaging is suitable for diagnosis in real time. In the case of low image quality, rescanning can be performed in the same examination time. This application may improve the workflow of examiners and reduce the patient burden.

Secondly, acoustic shadow detection is also a well-known US image preprocessing method. An acoustic shadow is one of the most representative artifacts, which is caused by several reflectors blocking the ultrasonic beams with rectilinear propagation from a transducer. Useful artifacts exist, such as the comet-tail artifact (B-line), which may provide diagnostic clues for COVID-19 infection in point-of-care lung US [37]. However, acoustic shadows are depicted in black with missing information in that region, and obstruct the examination and AI-based image recognition of the target organs in US imaging. Therefore, performing acoustic shadow detection prior to US imaging analysis may enable a judgment to be made on whether an acquired image is suitable as the input data. Traditional image processing methods for acoustic shadow detection include automatic geometrical and statistical methods using rupture detection of the brightness value along the scanning line [38], and random walk-based approaches [39,40]. In these methods, the parameters and models need to be carefully changed in response to a domain shift. However, deep learning-based methods can be applied to a wider range of domains. The preparation of the training dataset remains challenging as the pixel-level annotation of acoustic shadows is highly costly and difficult owing to their translucency and blurred boundaries. Meng et al. employed weakly supervised estimation of confidence maps using labels for each image with or without acoustic shadows [41,42]. Yasutomi et al. proposed a semi-supervised approach for integrating domain knowledge into a data-driven model using the pseudo-labeling of plausible synthetic shadows that were superimposed onto US imaging (Figure 1) [43].

## 3. Algorithms for US Imaging Analysis

In this section, we briefly present the fundamental machine learning algorithms for US imaging, along with other medical imaging modalities. Thereafter, we focus on specialized algorithms for US imaging analysis to overcome the noisy artifacts as well as the instability of the viewpoint and cross-section owing to manual operation.

**Figure 2:**
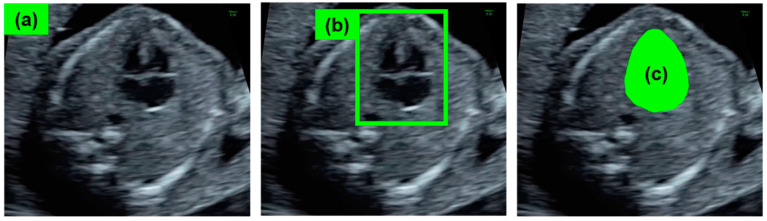
Fundamental algorithms generally used in US imaging analysis. (**a**) Image classification of whether the fetal US image contains a diagnostically useful cross-section such as a four-chamber view (4CV). (**b**) Detection of the fetal heart for evaluation of fetal heart structure. (**c**) Segmentation of the boundaries or regions of the fetal heart to measure the fetal cardiac index such as cardiothoracic area ratio (CTAR).

Classification, detection, and segmentation have generally been used as the fundamental algorithms in US imaging analysis (Figure 2). Classification estimates one or more labels for the entire image, and it has typically been used to seek the standard scanning planes for screening or diagnosis in US imaging analysis. ResNet [44] and Visual Geometry Group (VGG) [45] are examples of classification methods. Detection is mainly used to estimate lesions and anatomical structures. YOLO [46] and the single-shot multibox detector (SSD) [47] are popular detection algorithms. Segmentation is used for the further precise measurement of lesions and organ structures in pixels as well as index calculations of the lengths, areas, and volumes. U-Net [48] and DeepLab [49,50] are representative algorithms for segmentation. These standard algorithms are often used as baselines to evaluate the performance of specialized algorithms for US imaging analysis.

**Figure 3:**
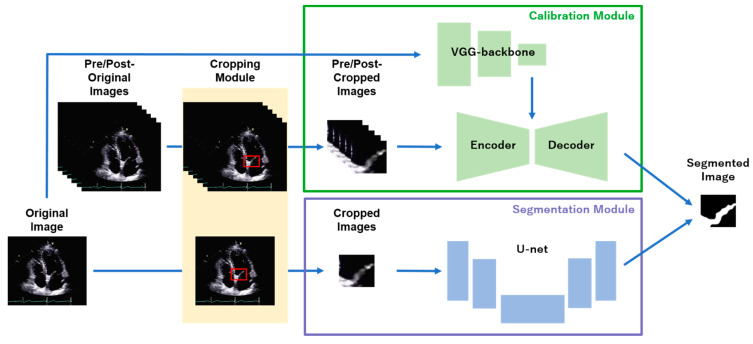
Use of time-series information to reduce noisy artifacts and to perform accurate segmentation in US videos. CSC employs the time-series information of US videos and specific section information to calibrate the output of U-Net [51].

We introduce the specialized algorithms for US imaging analysis to address the performance deterioration owing to noisy artifacts. Cropping–segmentation–calibration (CSC) [51] and the multi-frame + cylinder method (MFCY) [52] use time-series information to reduce noisy artifacts and to perform accurate segmentation in US videos (Figure 3). Deep attention networks have also been proposed for improved segmentation performance in US imaging, such as the attention-guided dual-path network [53] and a U-Net-based network combining a channel attention module and VGG [54]. A contrastive learning-based framework [55] and a framework based on the generative adversarial network (GAN) [56] with progressive learning have been reported to improve the boundary estimation in US imaging [57].

The critical issues resulting from the instability of the viewpoint and cross-section often become apparent when the clinical indexes are calculated using segmentation. One traditional US image processing method is the reconstruction of three-dimensional (3D) volumes [58]. Direct segmentation methods for conventional 3D volumes, including 3D U-Net [59], are useful for accurate volume quantification; however, their labeling is very expensive and time-consuming. The interactive few-shot Siamese network uses a Siamese network and a recurrent neural network to perform 3D segmentation training from few-annotated two-dimensional (2D) US images [60]. Another research subject is the extraction of 2D US images involving standard scanning planes from the 3D US volume. The iterative transformation network was proposed to guide the current plane towards the location of the standard scanning planes in the 3D US volume [61]. Moreover, Duque et al. proposed a semi-automatic segmentation algorithm for a freehand 3D US volume, which is a continuum of 2D cross-sections, by employing an encoder–decoder architecture with 2D US images and several 2D labels [62]. We summarize the abovementioned segmentation algorithms for US imaging analysis in Table 2.

## 4. Medical AI Research in US Imaging

### 4.1. Oncology

#### 4.1.1. Breast Cancer

Breast cancer is the most common cancer in woman globally [63]. US imaging is used extensively for breast cancer screening in addition to mammography. Various efforts have been made to date regarding the classification of benign and malignant breast tumors in US imaging. Han et al. trained the CNN model architecture to differentiate between benign and malignant breast tumors [64]. The Inception model, which is a CNN model with batch normalization, exhibited equivalent or superior diagnostic performance compared to radiologists [65]. Byra et al. introduced a matching layer to convert grayscale US images into RGB to leverage the discriminative power of the CNN more efficiently [66]. Antropova et al. employed VGG and the support vector machine for classification using the CNN features and conventional computer-aided diagnosis features [67]. A mass-level classification method enabled the construction of an ensemble network by combining VGG and ResNet to classify a given mass using all views [68]. Considering that both thyroid and breast cancers exhibit several similar high-frequency US characteristics, Zhu et al. developed a generic VGG-based framework to classify thyroid and breast lesions in US imaging [69]. The model that was constructed with features that were extracted from all three transferred models achieved the highest overall performance [70]. The Breast Imaging Reporting and Data System (BI-RADS) provides guidance and criteria for physicians to determine breast tumor categories based on medical images in clinical settings. Zhang et al. proposed a novel network that integrates the BI-RADS features into task-oriented semi-supervised deep learning for accurate diagnosis using US images with a small training dataset [71]. Huang et al. developed the ROI-CNN (ROI identification network) and the subsequent G-CNN (tumor categorization network) to generate effective features for classifying the identified ROIs into five categories [72]. The Inception model achieved the best performance in predicting lymph node metastasis from US images in patients with primary breast cancer [73].

Yap et al. investigated the use of three deep learning approaches for breast lesion detection in US imaging. The performances were evaluated on two datasets and the different methods achieved the highest performance for each dataset [74]. An experimental study was performed to evaluate the different CNN architectures on breast lesion detection and classification in US imaging, in which SSD for breast lesion detection and DenseNet [75] for classification exhibited the best performance [76].

Several ingenious segmentation methods for breast lesions in US imaging have been reported. Kumar et al. demonstrated the performance of the Multi-U-Net segmentation algorithm for suspicious breast masses in US imaging [77]. A novel automatic tumor segmentation method that combines a dilated fully convolutional network (FCN) with a phase-based active contour model was proposed [78]. Residual-dilated-attention-gate-U-Net is based on the conventional U-Net, but the plain neural units are replaced with residual units to enhance the edge information [79]. Vakanski et al. introduced attention blocks into the U-Net architecture to learn feature representations that prioritize spatial regions with high saliency levels [80]. Singh et al. proposed automatic tumor segmentation in breast US images using contextual-information-aware GAN architecture. The proposed model achieved competitive results compared to other segmentation models in terms of the Dice and intersection over union metrics [81].

#### 4.1.2. Thyroid Cancer

The incidence of thyroid cancer has been increasing globally as a result of overdiagnosis and overtreatment owing to the sensitive imaging techniques that are used for screening [82]. A CNN with the addition of a spatial constrained layer was proposed to develop a detection method that is suitable for papillary thyroid carcinoma in US imaging [83]. The Inception model achieved excellent diagnostic efficiency in differentiating between papillary thyroid carcinomas and benign nodules in US images. It could provide more accurate diagnosis of nodules that were 0.5 to 1.0 cm in size, with microcalcification and a taller shape [84]. Ko et al. designed CNNs that exhibited comparable diagnostic performance to that of experienced radiologists in differentiating thyroid malignancy in US imaging [85]. Furthermore, a fine-tuning approach based on ResNet was proposed, which outperformed VGG in terms of the classification accuracy of thyroid nodules [86]. Li et al. used CNNs for the US image classification of thyroid nodules. Their model exhibited similar sensitivity and improved specificity in identifying patients with thyroid cancer compared to a group of skilled radiologists [82].

#### 4.1.3. Ovarian Cancer

Ovarian cancer is the most lethal gynecological malignancy because it exhibits few early symptoms and generally presents at an advanced stage [87]. The screening methods for ovarian cysts using imaging techniques need to be improved to overcome the poor prognosis of ovarian cancer. Zhang et al. proposed an image diagnosis system for classifying ovarian cysts in color US images using the high-level deep features that were extracted by the fine-tuned CNN and the low-level rotation-invariant uniform local binary pattern features [88]. US imaging analysis using an ensemble model of CNNs demonstrated comparable diagnostic performance to human expert examiners in classifying ovarian tumors as benign or malignant [89].

#### 4.1.4. Prostate Cancer

Feng et al. presented a 3D CNN model to detect prostate cancer in sequential contrast-enhanced US (CEUS) imaging. The framework consisted of three convolutional layers, two sub-sampling pooling layers, and one fully connected classification layer. Their method achieved a specificity of over 91% specificity and an average accuracy of 90% over the targeted CEUS images for prostate cancer detection [90]. A random forest-based classifier for the multiparametric localization of prostate cancer lesions based on B-mode, shear-wave elastography, and dynamic contrast-enhanced US radiomics was developed [91]. A segmentation method was proposed for the clinical target volume (CTV) in the transrectal US image-guided intraoperative process for permanent prostate brachytherapy. A CNN was employed to construct the CTV shape in advance from automatically sampled pseudo-landmarks, along with an encoder–decoder CNN architecture for low-level feature extraction. This method achieved a mean accuracy of 96% and a mean surface distance error of 0.10 mm [92].

#### 4.1.5. Other Cancers

Hassan et al. developed stacked sparse auto-encoder and softmax classifier architecture for US image classification of focal liver diseases into a benign cyst, hemangioma, and hepatocellular carcinoma along with the normal liver [93]. Schmauch et al. proposed a deep learning model based on ResNet for the detection and classification of focal liver lesions into the abovementioned diseases, as well as focal nodular hyperplasia and metastasis in liver US images [94]. An ensemble model of CNNs was proposed for kidney US image classification into four classes, namely normal, cyst, stone, and tumor. This method achieved a maximum classification accuracy of 96% in testing with quality images and 95% in testing with noisy images [95].

### 4.2. Cardiovascular Medicine

#### 4.2.1. Cardiology

Echocardiography is the most common imaging modality in cardiovascular medicine, and it is frequently used for the screening as well as diagnosis and management of cardiovascular diseases [96]. Current technological innovations in echocardiography, such as the assessments of 3D US volumes and global longitudinal strain, are remarkable. Clinical evidence has been accumulating for the utilization of 3D echocardiography. However, 3D US volume is still inferior in spatial and temporal resolutions to 2D US images. To utilize these latest technologies, it is a prerequisite for examiners to have the skill levels of acquiring high-quality images in 2D echocardiography. In addition, echocardiography has become the primary point-of-care imaging modality for the early diagnosis of the cardiac symptoms of COVID-19 [97,98]. Therefore, it is expected that the clinical applications of AI will improve the diagnostic accuracy and workflow in echocardiography. To our knowledge, there is the highest number of the AI-powered medical devices for echocardiography among those devices which the FDA has been approved in application to US imaging.

Abdi et al. developed a CNN to reduce the user variability in data acquisition by automatically computing a score of the US image quality of the apical four-chamber view for examiner feedback [99]. Liao et al. proposed a quality assessment method for cardiac US images through modeling the label uncertainty in CNNs resulting from intra-observer variability in the labeling [100]. Deep learning-based view classification has also been reported. EchoNet could accurately identify the presence of pacemaker leads, an enlarged left atrium, and left ventricular (LV) hypertrophy by analyzing the local cardiac structures. In this study, the LV end systolic and diastolic volumes, and ejection fraction (EF), as well as the systemic phenotypes of age, sex, weight, and height, were also estimated [101]. Zhang et al. proposed a deep learning-based pipeline for the fully automated analysis of cardiac US images, including view classification, chamber segmentation, measurements of the LV structure and function, and the detection of specific myocardial diseases [102].

The assessment of regional wall motion abnormalities (RWMAs) is an important testing process in echocardiography, which can localize ischemia or infarction of coronary arteries. Strain imaging, including the speckle tracking method, has been used extensively to evaluate LV function in clinical practice. Ahn et al. proposed an unsupervised motion tracking framework using U-Net [103]. Kusunose et al. compared the area under the curve (AUC) obtained by several CNNs and physicians for detecting the presence of RWMAs. The CNN achieved an equivalent AUC to that of an expert, which was significantly higher than that of resident physicians [104].

#### 4.2.2. Angiology

Lekadir et al. proposed a CNN for extracting the optimal information to identify the different plaque constituents from carotid US images. The results of cross-validation experiments demonstrated a correlation of approximately 0.90 with the clinical assessment for the estimation of the lipid core, fibrous cap, and calcified tissue areas [105]. A deep learning model was developed for the classification of the carotid intima-media thickness to enable reliable early detection of atherosclerosis [106]. Araki et al. introduced an automated segmentation system for both the near and far walls of the carotid artery using grayscale US morphology of the plaque for stroke risk assessment [107]. A segmentation method that integrated the random forest and an auto-context model could segment the plaque effectively, in combination with the features extracted from US images as well as iteratively estimated probability maps [108]. The quantification of carotid plaques by measuring the vessel wall volume using the boundary segmentation of the media-adventitia (MAB) and lumen-intima (LIB) is sensitive to temporal changes in the carotid plaque burden. Zhou et al. proposed a semi-automatic segmentation method based on carotid 3D US images using a dynamic CNN for MAB segmentation and an improved U-Net for LIB segmentation [109]. Biswas et al. performed boundary segmentation of the MAB and LIB, incorporating a machine learning-based joint coefficient method for fine-tuning of the border extraction, to measure the carotid intima-media thickness from carotid 2D US images [110]. The application of a CNN and FCN to automated lumen detection and lumen diameter measurement was also presented [111]. The deep learning-based boundary detection and compensation technique enabled the segmentation of vessel boundaries by harnessing the CNN and wall motion compensation in the analysis of near-wall flow dynamics in US imaging [112]. Towards the cost-effective diagnosis of deep vein thrombosis, Kainz et al. employed a machine learning model for the detection and segmentation of the representative veins and the prediction of their vessel compression status [113].

### 4.3. Obstetrics

US imaging plays the most important role in medical diagnostic imaging in the obstetrics field. The non-invasiveness and real-time properties of US imaging enable fetal morphological and functional evaluations to be performed effectively. US imaging is used for the screening of congenital diseases, the assessment of fetal development and well-being, and the detection of obstetric complications [114]. Transvaginal US enables the clear observation of the fetus and other organs including the uterus, ovaries, and fallopian tubes, which are mainly located on the pelvic floor during the first trimester. Moreover, transabdominal US is useful for observing the fetal growth during the gestational weeks.

During fetal US imaging, numerous anatomical structures with small shapes and movement are simultaneously observed in clinical practice. Medical AI research has been conducted on the development of algorithms that are applicable to the US imaging analysis of the fetus or fetal appendages. Dozen et al. improved the segmentation performance of the ventricular septum in fetal cardiac US videos using cropped and original image information in addition to time-series information [51]. CSC can be applied to the segmentation of other organs that are small and have dynamically changing shapes with heartbeats, such as the heart valves. Shozu et al. proposed a novel model-agnostic method to improve the segmentation performance of the thoracic wall in fetal US videos. This method was based on ensemble learning of the time-series information of US videos and the shape information of the thoracic wall [52]. Medical AI research was conducted on the measurement of fetal anatomical segments in US imaging [115,116,117,118]. The scale attention pyramid deep neural network using multi-scale information could fuse local and global information to infer the skull boundaries that contained speckle noise or discontinuities. The elliptic geometric axes were modified by a regression network to obtain the fetal head circumference, biparietal diameter, and occipitofrontal diameter more accurately [119]. Kim et al. proposed a machine learning-based method for the automatic identification of the fetal abdominal circumference [120]. The localizing region-based active contour method, which was integrated with a hybrid speckle noise-reducing technique, was implemented for the automatic extraction and calculation of the fetal femur length [121]. A computer-aided detection framework for the automatic measurement of fetal lateral ventricles [122] and amniotic fluid volume [123] was also developed. The fully automated and real-time segmentation of the placenta from 3D US volumes could potentially enable the use of the placental volume to screen for an increased risk of pregnancy complications [124].

The acquisition of optimal US images for diagnosis in fetal US imaging is dependent on the skill levels of the examiners [4]. Therefore, it is essential to evaluate whether the acquired US images have a suitable cross-section for diagnosis. Furthermore, when labeling a huge amount of US images for AI-based image processing, it is necessary to classify the acquired US images and to assess whether the image quality thereof is suitable for the input data. Burgos-Artizzu et al. evaluated a wide variety of CNNs for the automatic classification of a large dataset containing over 12,400 images from 1792 patients that were routinely acquired during maternal-fetal US screening [125]. An automatic recognition method using deep learning for the fetal facial standard planes, including the axial, coronal, and sagittal planes was reported [126]. Moreover, automated partitioning and characterization on an unlabeled full-length fetal US video into 20 anatomical or activity categories was performed [127]. A generic deep learning framework for the automatic quality control of fetal US cardiac four-chamber views [128] as well as a framework for tracking the key variables that described the contents of each frame of freehand 2D US scanning videos of a healthy fetal heart [129] were developed. Wang et al. presented a deep learning framework for differentiating operator skills during fetal US scanning using probe motion tracking [130].

**Figure 4:**
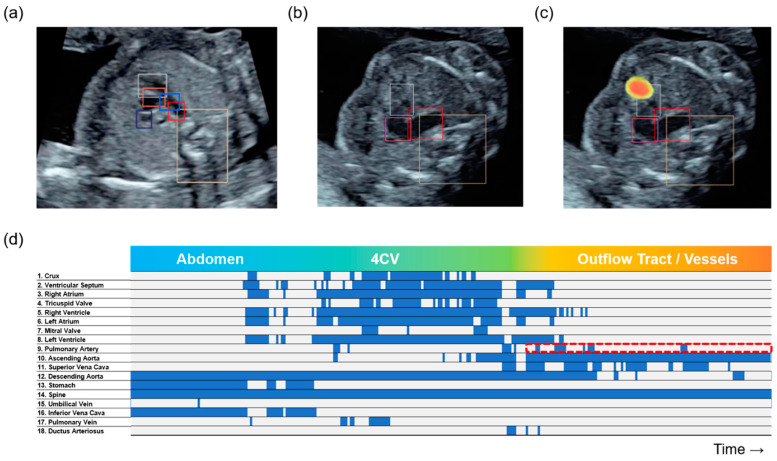
Possible techniques for AI explainability. The cardiac substructures were detected with colored bounding boxes in a three-vessel trachea view in (**a**) a normal case, and (**b**) a tetralogy of Fallot (TOF) case. (**c**) An image of the class-specific heatmap indicates the discriminative regions of the image that caused the particular class activity of interest. (**d**) Barcode-like timeline in a TOF case. The vertical axis represents the 18 selected substructures and the horizontal axis represents the examination timeline in the rightward direction. A probability of ≥0.01 was set as well-detected and is indicated as the blue bar, whereas <0.01 was set as non-detected and is indicated by the gray bar in each frame. The pulmonary artery was not detected (red dotted box).

AI-based abnormality detection and classification in fetal US imaging remain challenging owing to the wide variety and relatively low incidence of congenital diseases. Xie et al. proposed deep learning algorithms for the segmentation and classification of normal and abnormal fetal brain US images in the standard axial planes. Furthermore, they provided heat maps for lesion localization using gradient-weighted class activation mapping [131]. An ensemble of neural networks, which was trained using 107,823 images from 1326 retrospective fetal cardiac US studies, could identify the recommended cardiac views as well as distinguish between normal hearts and complex congenital heart diseases. Segmentation models were also proposed to calculate standard fetal cardiothoracic measurements [132]. Komatsu et al. proposed the CNN-based architecture known as supervised object detection with normal data only (SONO) to detect 18 cardiac substructures and structural abnormalities in fetal cardiac US videos. The abnormality score was calculated using the probability of the cardiac substructure detection. SONO enables abnormalities to be detected based on the difference from the correct anatomical localization of normal structures, thereby addressing the challenge of the low incidence of congenital heart diseases. Furthermore, in our previous work, the above probabilities were visualized similar to a barcode-like timeline. This timeline was useful in terms of AI explainability when detecting cardiac structural abnormalities in fetal cardiac US videos (Figure 4) [133].

Deep learning-incorporated software improved the prediction performance of neonatal respiratory morbidity induced by respiratory distress syndrome or transient tachypnea of the newborn in fetal lung US imaging for AI-based fetal functional evaluation [134].

## 5. Discussion and Future Directions

In this review, we have introduced various areas of medical AI research with a focus on US imaging analysis to understand the global trends and future research subjects from both the clinical and basic perspectives. In addition to other medical imaging modalities, classification, detection, and segmentation are the fundamental tasks of AI-based image analysis. However, US imaging exhibits several issues in terms of image quality control. Thus, US image preprocessing needs to be performed and ingenious algorithm combinations are required.

Acoustic shadow detection is the characteristic task in US imaging analysis. Although deep learning-based methods can be applied to a wide range of domains, the preparation of training datasets remains challenging. Therefore, weakly or semi-supervised methods offer the advantage of cost-effectiveness for labeling [41,42,43]. Towards the clinical application of acoustic shadow detection methods, examiners can evaluate whether the current acquired US imaging is suitable for diagnosis in real time. If not, rescanning can be performed during the same examination time. This application may improve the workflow of examiners and reduce the patient burden. Several frameworks relating to specialized algorithms for US imaging analysis have been proposed, in which the time-series information in US video [51,52] or a channel attention module [53,54] have been integrated with conventional algorithms to overcome the performance deterioration owing to noisy artifacts. Furthermore, the AI-based analysis of 3D US volumes is expected to resolve the problem of the viewpoint and cross-section instability resulting from manual operation.

From a clinical perspective, breast cancer and cardiovascular diseases are medical fields in which substantial research efforts in AI-based US imaging analysis have been made to date, resulting in more medical AI devices being approved. Considering the clinical background of these two medical fields in which US imaging is commonly used, the potential exists to develop medical AI research and technologies in obstetrics as well. However, AI-based US imaging analysis remains challenging and few medical AI devices are available for this purpose. Therefore, deep learning-based methods that are applicable to cross-disciplinary studies and a wide range of domains need to be learned and incorporated. According to our review, several ingenious segmentation methods for target lesions or structures in US imaging may apply to cross-disciplinary utilization among oncology, cardiovascular medicine, and obstetrics. For example, CSC can be applied to the segmentation of other small and deformable organs using time-series information of US videos. Valid US diagnostic support technologies can be established in clinical practice by accumulating AI-based US image analyses. Automated image quality assessment and detection can lead to the development of a scanning guide and training material for examiners. Accurate volume quantification as well as the measurement of lesions and indexes can result in an improved workflow and a reduction in examiner bias. AI-based abnormality detection is expected to be used for the objective evaluation of lesions or abnormalities and in preventing oversights. However, it remains challenging to prepare sufficient datasets on both normal and abnormal subjects for the target diseases. To address the data preparation issue, it is possible to implement AI-based abnormality detection using correct anatomical localization and the morphologies of normal structures as a baseline [133].

Furthermore, AI explainability is key to the clinical application of AI-based US diagnostic support technologies. It is necessary for examiners to understand and explain their rationale for diagnosis to patients when obtaining informed consent. Class activation mapping is a popular technique for AI explainability, which enables the computation of class-specific heatmaps indicating the discriminative regions of the image that caused the particular class activity of interest [135]. Zhang et al. provided an interpretation for regression saliency maps, as well as an adaptation of the perturbation-based quantitative evaluation of explanation methods [136]. ExplainGAN is a generative model that produces visually perceptible decision-boundary crossing transformations, which provide high-level conceptual insights that illustrate the manner in which a model makes decisions [137]. We proposed a barcode-like timeline to visualize the progress of the probability of substructure detection along with sweep scanning in US videos. This technique was demonstrated to be useful in terms of AI explainability when we detected cardiac structural abnormalities in fetal cardiac US videos. Moreover, the barcode-like timeline diagram is informative and understandable, thereby enabling examiners of all skill levels to consult with experts knowledgeably [133].

Towards the clinical application of medical AI algorithms and devices, it is important to understand the approval processes and regulations of the US FDA, the Japan Pharmaceuticals and Medical Devices Agency, and the responsible institutions of other countries. Furthermore, knowledge of the acts on the protection of personal information and the guidelines for handling all types of medical data, including the clinical information of patients and medical imaging data, should be updated. Wu et al. compiled a comprehensive overview of medical AI devices that are approved by the FDA and pointed out the limitations of the evaluation process that may mask the vulnerabilities of devices when they are developed on patients [25]. In the majority of evaluations, only retrospective studies have been performed. These authors recommended the performance evaluation of medical AI devices in multiple clinical sites, prospective studies, and post-market surveillance. Moreover, industry–academia–medicine collaboration is required to share valuable concepts in the development of medical AI devices for patients and examiners, and its actual use in clinical practice.

The utilization of AI and internet of things (IoT) technologies, along with advanced networks such as 5G, will presently accelerate infrastructure development in the medical field, including remote medical care and regional medical cooperation. The current COVID-19 pandemic has also provided an opportunity to promote such developments. US imaging is the most common medical imaging modality in an extensive range of medical fields. However, stronger support for examiners in terms of image quality control should be considered. The clinical implementation of AI-based US diagnostic support technologies is expected to correct the medical disparities between regions through examiner training or by remote diagnosis using cloud-based systems.

## Figures and Tables

**Table 1:** List of FDA-approved medical AI devices for US imaging.

No.	FDA Approval Number	Product Name/Company	Description	Body Area	Decision Date	Regulatory Class/Submission Type
1	K161959	ClearView cCAD/ClearView Diagnostics, Inc., Piscataway, NJ, USA	Automatically classifies the shape and orientation characteristics of user-selected ROIs in breast US images with the BI-RADS category using machine learning.	Breast	28 December 2016	Class II/510(k)
2	K162574	AmCAD-US/AmCAD BioMed Corporation, Taipei, Taiwan	Visualizes and quantifies US image data with backscattered signals echoed by tissue compositions.	Thyroid	30 May 2017	Class II/510(k)
3	K173780	EchoMD AutoEF software/Bay Labs, Inc., San Francisco, CA, USA	Provides automated estimation of the LVEF on previously acquired cardiac US images using machine learning.	Heart	14 June 2018	Class II/510(k)
4	K180006	AmCAD-UT Detection 2.2/AmCad BioMed Corporation, Taipei, Taiwan	Analyzes thyroid US images of user-selected ROIs. Provides detailed information with the quantification and visualization of US characteristics of thyroid nodules.	Thyroid	31 August 2018	Class II/510(k)
5	K190442	Koios DS/Koios Medical, Inc., New York, NY, USA	Diagnostic aid using machine learning to characterize US image features with user-provided ROIs to generate categorical output that aligns to BI-RADS and the auto-classified shape and orientation.	Breast	3 July 2019	Class II/510(k)
6	K191171	EchoGo Core/Ultromics Ltd., Oxford, UK	Automatically measures cardiac US parameters including EF, Global Longitudinal Strain, and LV volume using machine learning.	Heart	13 November 2019	Class II/510(k)
7	DEN190040	Caption Guidance/Caption Health, Inc., Brisbane, CA, USA	Assists in the acquisition of anatomically correct cardiac US images that represent standard 2D echocardiographic diagnostic views and orientations using deep learning.	Heart	7 February 2020	Class II/De Novo
8	K200356	MEDO ARIA/Medo.ai, Inc., Edmonton, Canada	Views and quantifies US image data to aid trained medical professionals in the diagnosis of developmental dysplasia of the hip using machine learning.	Hip	11 June 2020	Class II/510(k)
9	K200980	Auto 3D Bladder Volume Tool/Butterfly Network, Inc., Guilford, CT, USA	Views, quantifies, and reports the results acquired on Butterfly Network US systems using machine learning-based 3D volume measurements of the bladder.	Bladder	11 June 2020	Class II/510(k)
10	K200621	Caption Interpretation Automated Ejection Fraction Software/Caption Health, Inc., Brisbane, CA, USA	Processes previously acquired cardiac US images and provides machine learning-based estimation of the LVEF.	Heart	22 July 2020	Class II/510(k)
11	K201369	AVA (Augmented Vascular Analysis)/See-Mode Technologies Pte. Ltd., Singapore, Singapore	Analyzes vascular US scans including vessel wall segmentation and measurement of the intima-media thickness of the carotid artery using machine learning.	Carotid artery	16 September 2020	Class II/510(k)
12	K201555	EchoGo Pro/Ultromics Ltd., Oxford, UK	Decision support system for diagnostic stress ECG using machine learning to assess the severity of CAD using LV segmentation of cardiac US images.	Heart	18 December 2020	Class II/510(k)
13	K210053	LVivo software application/DiA Imaging Analysis Ltd., Beer Sheva, Israel	Evaluates the LVEF using deep learning-based LV segmentation in cardiac US images.	Heart	5 February 2021	Class II/510(k)

Abbreviations: ROI, region of interest; BI-RADS, Breast Imaging Reporting and Data System; LVEF, left ventricular ejection fraction; ECG, echocardiography; CAD, coronary artery disease.

**Table 2:** List of segmentation algorithms for US imaging analysis.

Algorithm Name	Description	Ref.
U-Net	Based on a fully convolutional network and achieves more accurate segmentation using smaller amounts of training data compared with the other methods. Several studies have reported superior segmentation performances using their models based on U-Net, which is particularly suitable for biomedical image segmentation.	[48]
DeepLab	Utilizes atrous convolution and demonstrates its state-of-the-art segmentation performance. DeepLabv3+ is the latest version developed by combining pyramidal pooling modules with an encoder-decoder model.	[49,50]
CSC	Utilizes time-series information to reduce noisy artifacts and performs accurate segmentation on a small and deformable organ in US videos.	[51]
MFCY	Uses time-series information and demonstrates high-performance segmentation on a target organ with a cylindrical shape in US videos.	[52]
AIDAN	The attention-guided dual-path network improves segmentation performance in US imaging.	[53]
Deep attention network	A U-Net-based network combining a channel attention module and VGG improves segmentation performance in US imaging.	[54]
Contrastive rendering	A contrastive learning-based framework improves the boundary estimation in US imaging.	[55]
GAN-based method	A GAN-based framework with progressive learning improves the boundary estimation in US imaging.	[57]
3D U-Net	The representative direct segmentation method for conventional 3D volumes is useful for accurate volume quantification.	[59]
IFSS-NET	The interactive few-shot Siamese network uses a Siamese network and a recurrent neural network to perform 3D segmentation training from few-annotated 2D US images.	[60]
Encoder–decoder architecture	A semi-automatic segmentation algorithm for a freehand 3D US volume by employing an encoder–decoder architecture with 2D US images and several 2D labels.	[62]

Abbreviations: CSC, cropping–segmentation–calibration; MFCY, multi-frame + cylinder method; AIDAN, attention-guided dual-path network; GAN, generative adversarial network; IFSS-NET, interactive few-shot Siamese network.

## Data Availability

Data sharing is not applicable owing to the patient privacy rights.
